# A New Methodological Approach to the Characterization of Optimal Charging Rates at the Hydrogen Plasma Smelting Reduction Process Part 1: Method

**DOI:** 10.3390/ma15144767

**Published:** 2022-07-07

**Authors:** Michael Andreas Zarl, Daniel Ernst, Julian Cejka, Johannes Schenk

**Affiliations:** 1 Department of Metallurgy, Chair of Ferrous Metallurgy, Montanuniversität Leoben, Franz-Josef Straße 18, 8700 Leoben, Austria; daniel.ernst@unileoben.ac.at (D.E.); julian.cejka@unileoben.ac.at (J.C.); johannes.schenk@unileoben.ac.at (J.S.); 2 K1-MET GmbH, Stahlstraße 14, 4020 Linz, Austria

**Keywords:** arc plasma, arc stability, smelting reduction, iron ore, hydrogen, reduction, thermal plasma, hydrogen-based metallurgy

## Abstract

The development of a carbon lean steel production process following the concept of direct carbon avoidance is one of the most challenging tasks the iron and steel industry must tackle in just a few decades. The necessary drastic reduction of 80% of the process’s inherent emissions by 2050 is only possible if a new process concept that uses hydrogen as the primary reductant is developed. The Hydrogen Plasma Smelting Reduction (HPSR) of ultra-fine iron ores is one of those promising concepts. The principle was already proven at a lab scale. The erection of a bench-scale facility followed this, and further scaled-ups are already planned for the upcoming years. For this scale-up, a better understanding of the fundamentals of the process is needed. In particular, knowledge of the kinetics of the process is essential for future economically feasible operations. This investigation shows the principles for evaluating and comparing the process kinetics under varying test setups by defining a representative kinetic parameter. Aside from the fundamentals for this definition, the conducted trials for the first evaluation are shown and explained. Several differences in the reduction behavior of the material at varying parameters of the process have already be shown. However, this investigation focuses on the description and definition of the method. An overall series of trials for detailed investigations will be conducted as a follow-up.

## 1. Introduction

The Hydrogen Plasma Smelting Reduction Process (HPSR) development at the Chair of Ferrous Metallurgy in Leoben has a long history. The process’s basic engineering has been developing since 1992, and the research is not yet finished [1,2,3,4,5]. The HPSR process is a one-step process for producing steel using ultra-fine ore and is a potential new approach for low-carbon steel production. One of the biggest challenges for the European Union is to reach its zero-carbon target by 2050 and halve emissions by 2030. Therefore, European steel producers have been challenged with quickly becoming green steel producers [6,7,8,9,10,11,12,13].

For this reason, groundbreaking new technologies that apply the principle of direct carbon avoidance are urgently needed. The development of the HPSR process has reached a critical point where the basic principle has already proven itself [14,15,16,17,18,19,20,21,22]. Still, the scale-up to the final industrial plant has only just begun. Further additional information must be collected for this scale-up, and many further analyses must be performed to find systems that allow for a comparison between the development steps. A fundamental study on the arc’s stability has already been carried out [23]. In addition to the arc’s stability, the rate of ore-charging and the connected kinetic parameters are some of the most critical issues to be investigated. This investigation focuses on defining a method for evaluating and comparing the optimal charging rates for the process. This method identifies the point where the parameters for reduction facilitate the highest rate for transforming solid ore into liquid steel. Therefore, the batch quantity, the liquid slag and steel bath conditions at the beginning of charging, and the charging time must be evaluated. This method defines the influences of the optimal kinetics for the process. The research shown in this study functions as a basis for further process development and is one of many steps in the development of the HPSR process at an industrial scale.

## 2. Arc Plasma Basics for the HPSR Process

This section explains the stated findings’ essential theoretical facts. After a short introduction to the thermodynamics and kinetics of hydrogen reduction, an equation for the description of the reduction rate of the process is displayed. The method for identifying the optimal charging rate (described afterwards) is based on this approach. Further information concerning the theoretical background of the thermodynamics and kinetics can be found in former publications [24,25,26,27,28,29].

### 2.1. HPSR Basics of Thermodynamics and Kinetics

In the HPSR process, different types of hydrogen exist simultaneously. Depending on the arc plasma flame’s thermal profile, they occur in different plasma gas concentrations. Equations (1)–(5) show hydrogen plasmas’ primary dissociation, ionization, and recombination types. Additionally, H_2_^+^, H_3_^+^, and the rovibrational state of the hydrogen molecule H_2_* can occur.

(1) $$e^{-}+H_{2}\overset{}{\to }2H+e^{-}\;\mathrm{collisional}\;\mathrm{dissociation}$$

(2) $$e^{-}+H\overset{}{\to }H^{+}+2e^{-}\;\mathrm{collisional}\;\mathrm{ionization}$$

(3) $$h\nu +H\overset{}{\to }H^{+}+e^{-}\;\mathrm{photoionization}$$

(4) $$H^{+}+2e^{-}\overset{}{\to }H+e^{-}\;\mathrm{three}-\mathrm{body}\;\mathrm{recombination}$$

(5) $$H^{+}+e^{-}\overset{}{\to }H\;\mathrm{two}-\mathrm{body}\;\mathrm{recombination}$$

According to Zhang et al. [30], the hydrogen species’ reduction potential can be arranged from high to low.

H^+^ > H_2_^+^ > H_3_^+^ > H > H_2_* > H_2_

**Figure 1:**
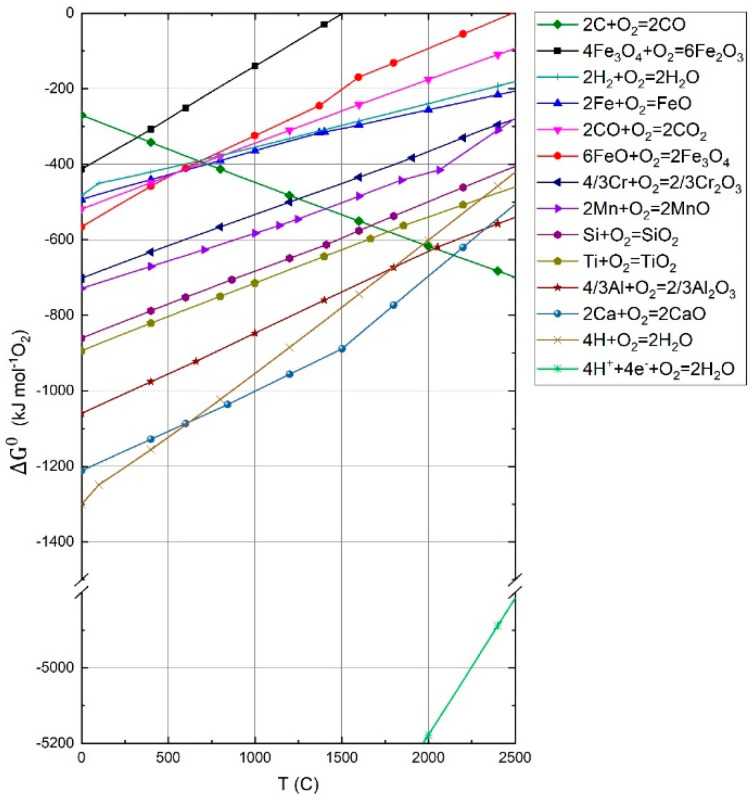
Ellingham–Richardson diagram for various metal oxides calculated using FactSage^TM^ 8.0, Database FToxide (2019) [5].

The actual reduction potential of hydrogen in the plasma state can be explained using the Richardson–Ellingham diagram. Figure 1 shows the Ellingham diagram calculated by FactSage 8.0 (GGT, Herzogenrath, Germany) with additional hydrogen lines in an atomized and ionized form. The chart shows the Gibbs free energy of the metals’ reaction with one mole of oxygen via the temperature. All metal and oxides activities are set to 1, and all reactions are standardized with one mole of molecular oxygen. In this particular case, the *y*-axis could also be interpreted as the oxygen potential due to the relationship in Equation (6). The lower the ΔG value of a chemical reaction, the more stable the product is, which means that the lower the reaction line is set in the diagram, the higher the reduction potential of the metal, or in the case of carbon monoxide and hydrogen, of the gas. To obtain information about the ΔG value of the final reaction, a combination of two or more reactions listed in the diagram can be performed using Hess’s law. An example of such a combination can be seen in Equation (11), which combines Equations (7)–(10) to reduce iron oxides to metallic iron with molecular hydrogen.

(6) $$\Delta G^{0}=\mathrm{RTln}\left(p_{O_{2}}\right)$$

(7) $$4{\mathrm{Fe}}_{3}O_{4}\left(s\right)+O_{2}\left(g\right)\overset{\;}{\Rightarrow }6{\mathrm{Fe}}_{2}O_{3}\left(s\right)\;{\Delta G}_{298}=-406.7\;\mathrm{kJ}/\mathrm{mol}$$

(8) $$6\mathrm{FeO}\left(s\right)+O_{2}\left(g\right)\overset{\;}{\Rightarrow }2{\mathrm{Fe}}_{3}O_{4}(s)\;{\Delta G}_{298}=-559.4\;\mathrm{kJ}/\mathrm{mol}$$

(9) $$2\mathrm{Fe}\left(s\right)+O_{2}\left(g\right)\overset{\;}{\Rightarrow }2\mathrm{FeO}(s)\;{\Delta G}_{298}=-489.7\;\mathrm{kJ}/\mathrm{mol}$$

(10) $$2H_{2}\left(g\right)+O_{2}\left(g\right)\overset{\;}{\Rightarrow }2H_{2}O(g)\;{\Delta G}_{298}=-457.2\;\mathrm{kJ}/\mathrm{mol}$$

(11) $${\mathrm{Fe}}_{2}O_{3}\left(s\right)+3H_{2}\left(g\right)\overset{\;}{\Rightarrow }2\mathrm{Fe}\left(s\right)+3H_{2}O\left(g\right)\;{\Delta G}_{298}=58.2\;\mathrm{kJ}/\mathrm{mol}$$

The final equation shows that the reduction with molecular hydrogen is endergonic. This situation changes drastically if the molecular hydrogen is replaced by atomic or ionized hydrogen. Equations (12) and (13) show the Gibbs free energy for either reaction at 298 K. For the final reduction reactions given in Equations (14) and (15), the Gibbs free energy indicates a strongly exergonic behavior for the reduction.

(12) $$4H\left(g\right)+O_{2}\left(g\right)\overset{\;}{\Rightarrow }2H_{2}O\;{\Delta G}_{298}=-1270.4\;\mathrm{kJ}/\mathrm{mol}$$

(13) $$4H^{+}\left(g\right)+O_{2}\left(g\right)+4e^{-}\overset{\;}{\Rightarrow }2H_{2}O\;{\Delta G}_{298}=-6472.5\;\mathrm{kJ}/\mathrm{mol}$$

(14) $${\mathrm{Fe}}_{2}O_{3}\left(s\right)+6H\left(g\right)\overset{\;}{\Rightarrow }2\mathrm{Fe}\left(s\right)+3H_{2}O\left(g\right)\;{\Delta G}_{298}=-1161.6\;\mathrm{kJ}/\mathrm{mol}$$

(15) $${\mathrm{Fe}}_{2}O_{3}\left(s\right)+6H^{+}\left(g\right)+6e^{-}\overset{\;}{\Rightarrow }2\mathrm{Fe}\left(s\right)+3H_{2}O\left(g\right)\;\;{\Delta G}_{298}=-8964.7\;\mathrm{kJ}/\mathrm{mol}$$

Compared to molecular hydrogen, atomic and ionized hydrogen’s oxygen potential is 3 and 15 times higher [1,4,31]. This enormous difference in potential means that both species can reduce the lowest calcium line, which has the highest oxygen affinity. Nevertheless, the hydrogen concentration in the atomic and ionized states is relatively low in the entire process. Although the plasma gas mixture’s reduction potential is high enough to reduce iron, it is insufficient to reduce all metals. Previous studies show that SiO leaves the reaction chamber vaporized, but no silicon is detectable in the final product [2,3,4,32].

**Figure 2:**
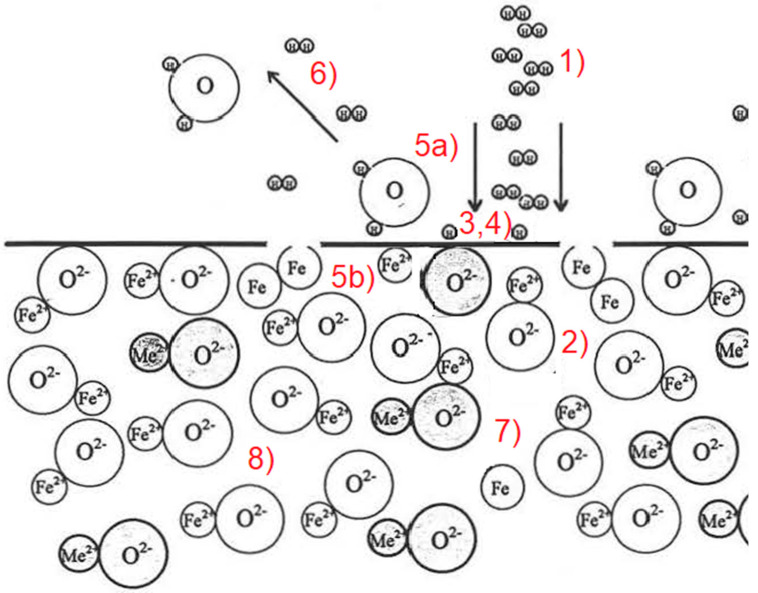
Necessary steps for hydrogen plasma reaction with a slag phase containing iron oxide [2].

The reduction occurs due to the thermodynamically favorable conditions in the HPSR process. Kamiya et al. [33] and Bäck [2] describe the steps necessary for a hydrogen plasma reaction with a slag phase containing iron oxide (Figure 2).

1. Transport of hydrogen in a molecular, excited, or ionized state to the oxide melt through the boundary layer, consisting of reaction products and inert gases, to the phase interface between the plasma gas and slag phase.
2. The transportation of the iron oxides of the slag to the phase boundary through the boundary layer may consist of accompanying oxides. Furthermore, solid precipitation due to an increased liquidus temperature or build-up of iron can have an inhibitory effect.
3. Adsorption of molecular or atomic hydrogen and oxygen of the dissociated iron oxides at the phase boundary.
4. The chemical reaction at the phase boundary.
5. Desorption of water vapor (5a) and metallic iron (5b) from the phase boundary.
6. Transport of water vapor through the boundary layer into the gas phase.
7. The higher density usually drives the removal of the reduced iron compared to the oxide melt.
8. The removal of the accompanying oxides, which often have a high melting point (CaO, SiO_2_, and Al_2_O_3_), and can therefore cause an increase in the liquidus temperature when the FeO content decreases, unless they are removed from the reaction site by the flow.

According to the statements of Sormann [1], the chemical reactions for these steps are those listed in Equations (16)–(19)

(16) $$H_{2}↔2H^{+}+2e^{-}$$

(17) $$H^{+}+O^{2-}↔{\mathrm{OH}}^{-}$$

(18) $$2{\mathrm{OH}}^{-}↔O^{2-}+H_{2}O$$

(19) $${\mathrm{Fe}}^{2+}+2e^{-}↔\mathrm{Fe}$$

These formulas are summarized in Equations (20)–(22) for the individual reactions of hematite, magnetite, and wüstite towards iron to describe the chemical reactions during the reduction of iron oxides using hydrogen plasma [24].

(20) $$3\left({\mathrm{Fe}}_{2}O_{3}\right)+\left\{\mathrm{Hydrogen}\;\mathrm{plasma}\;\left(2H,2H^{+},H_{2}^{+},\frac{2}{3}H_{3}^{+},H_{2}^{*}\right)\right\}↔2\left({\mathrm{Fe}}_{3}O_{4}\right)+\left\{H_{2}O\right\}$$

(21) $$\left({\mathrm{Fe}}_{2}O_{3}\right)+\left\{\mathrm{Hydrogen}\;\mathrm{plasma}\;\left(2H,2H^{+},H_{2}^{+},\frac{2}{3}H_{3}^{+},H_{2}^{*}\right)\right\}↔2\left(\mathrm{FeO}\right)+\left\{H_{2}O\right\}$$

(22) $$\left(\mathrm{FeO}\right)+\left\{\mathrm{Hydrogen}\;\mathrm{plasma}\;\left(2H,2H^{+},H_{2}^{+},\frac{2}{3}H_{3}^{+},H_{2}^{*}\right)\right\}↔\left[\mathrm{Fe}\right]+\left\{H_{2}O\right\}$$

The time-dependent conversion during the hydrogen reduction of solid iron oxides is often described by the following Equations (23)–(25). For the equilibrium constants of these equations, the chemical reactions are shown in Formulas (26)–(28) [34].

(23) $$\frac{dn_{h}}{dt}=-3n_{h}S_{h}k_{0}^{h}exp\left(-\frac{E_{h}}{RT}\right)\left(c^{H_{2}}-\frac{c^{H_{2}O}}{K_{h}}\right)\;$$

(24) $$\frac{dn_{m}}{dt}=-n_{m}S_{m}k_{0}^{m}exp\left(-\frac{E_{m}}{RT}\right)\left(c^{H_{2}}-\frac{c^{H_{2}O}}{K_{m}}\right)\;$$

(25) $$\frac{dn_{w}}{dt}=-n_{w}S_{w}k_{0}^{w}exp\left(-\frac{E_{w}}{RT}\right)\left(c^{H_{2}}-\frac{c^{H_{2}O}}{K_{w}}\right)\;$$

$n_{h}$, $n_{m}$, $n_{w}$—Quantity of hematite/magnetite/wüstite [mol_h/m/w_]

$S_{h}$, $S_{m}$, $S_{w}$—Specific surface [m^2^/mol]

$k_{0}^{h}$, $k_{0}^{m}$, $k_{0}^{w}$—Pre-exponential factor [m/s]

$E_{h}$, $E_{m}$, $E_{w}$—Activation energy [kJ/mol]

*R*—Ideal gas constant [kJ/mol*K]

*t*—Time [s]

*T*—Reaction temperature [K]

$c^{H_{2}}$, $c^{H_{2}O}$—Concentration of hydrogen/water in the gas [mol/m^3^]

$K_{h}$, $K_{m}$, $K_{w}$—Equilibrium constants of Formulas (26) to (28)

(26) $$3{\mathrm{Fe}}_{2}O_{3}+H_{2}↔2{\mathrm{Fe}}_{3}O_{4}+H_{2}O$$

(27) $$2{\mathrm{Fe}}_{3}O_{4}+2H_{2}↔\mathrm{FeO}+2H_{2}O$$

(28) $$\mathrm{FeO}+H_{2}↔\mathrm{Fe}+H_{2}O$$

**Figure 3:**
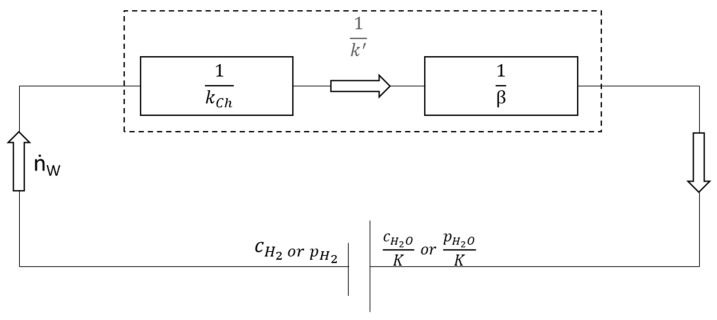
Schematic for the reduction of wüstite with hydrogen. Shown as a replacement diagram for the resistance network of the total reaction considering chemical reaction and mass transport.

Equations (23)–(25) describe the iron oxide’s reduction kinetics if the chemical reaction is limiting the reaction rate. The mass transport can become the rate-determining step in a reaction involving different phases, e.g., plasma and liquid phases. Consequently, the definition of the rate constant must be extended, and a mass transport coefficient (β) must be introduced. Figure 3 represents an analogous electrical scheme for this circumstance. The potential in the circuit diagram is the concentration difference of hydrogen to the water content at equilibrium. The resistances are in series and represent the rate constant of the chemical reaction ($\frac{1}{k_{Ch}}$) and mass transport coefficient ($\frac{1}{\beta }$). The corresponding equations are shown in Equations (29)–(32). Nagasaka et al. [35] formulated this generally for pure wüstite in liquid form as follows (Equation (33)). This equation is valid for reducing a liquid iron oxide melt with hydrogen plasma. The equation simplifies the system by combining $\left(S_{w}k_{0}^{w}\exp\left(-\frac{E_{w}}{RT}\right)\right)$ in one substitutional factor (k_a_). The reaction rate corresponds to the formation of water. Therefore, the rate of oxygen production results in a positive value [36] pp. 371–373.

(29) $$\frac{dn_{O}}{dt}=k_{a}\left(p_{H_{2}}-\frac{p_{H_{2}O}}{K}\right)$$

(30) $$k_{a}=k^{\prime }*\frac{M_{O}}{M_{FeO}}*S_{W}$$

(31) $$\frac{1}{k^{\prime }}=\frac{1}{k_{Ch}}+\frac{1}{\beta }$$

(32) $$k_{Ch}=k_{0}*e^{\left(-\frac{EA}{RT}\right)}$$

(33) $$r=k_{a}\left[H_{2}\right]\left(P_{H_{2}}-\frac{P_{H_{2}O}}{K_{H}^{\prime }}\right)$$

*r*—Reaction rate [kg oxygen/m^2^s]

*k_a_*[H_2_]—Reaction rate constant wüstite/H_2_ [kg oxygen/m^2^ s Pa]

$P_{H_{2}}$, $P_{H_{2}O}$—Partial pressure of hydrogen/water [Pa]

$K_{H}^{\prime }$—Partial pressure ratio (^p^_H_2_O_)/(^p^_H_2__) in equilibrium

The following Equation (34) is proposed for a temperature of 1673 K of the molten slag phase consisting only of FeO [35].

(34) $$r=1.6*10^{-6}\left(P_{H_{2}}-\frac{P_{H_{2}O}}{K_{H}^{\prime }}\right)$$

### 2.2. Aim of the Work Shown

The reduction of iron ore with hydrogen plasma is theoretically well described but remains a complex system to analyze. Multiple phases and reactions for the reduction need to be considered. The reduction in the bath, the ionization of the gas upon changing the geometry of the arc, the introduction of the material, and the phase of the material transition from the gas stream to the bath are just a few examples of the multiple influences that need to be considered. The definition of the charging parameters for the optimal kinetics is especially difficult but essential for the economic feasibility of the process. Therefore, a method for the characterization and comparison of representative reduction rates at varying setups of charging parameters needs to be defined. The technique should compare multiple parameter variations and their influences on the reduction rate. The main influencing parameters that can be directly set are the charging rate, the amount of charged material, and the pre-charged batch of the material before charging.

## 3. Materials and Methods

### 3.1. HPSR Laboratory Equipment

**Figure 4:**
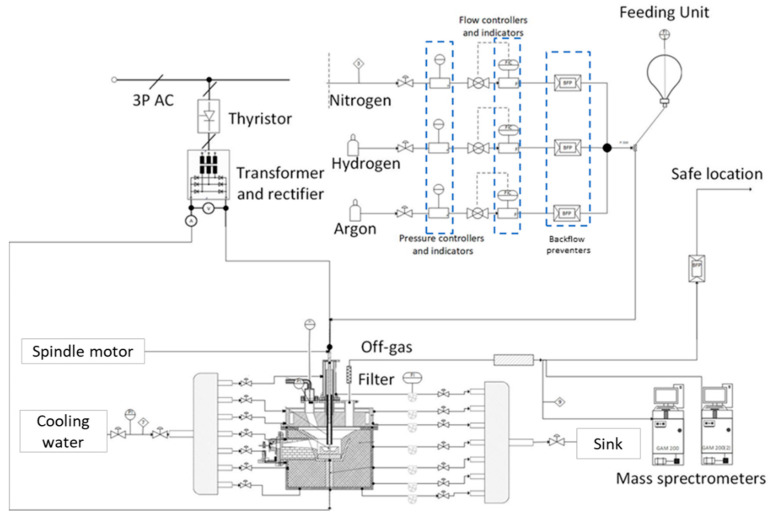
Layout of the HPSR laboratory facilities at the Chair of Ferrous Metallurgy.

The HPSR laboratory plant at Montanuniversitaet Leoben, Austria, was used. The facilities have been described multiple times in previous works. In the most recent article about arc stability fields, all facilities were described in detail [23]. Nevertheless, the equipment and the flowsheet of the process are summarized in Table 1 and Figure 4.

### 3.2. Experimental Program and Materials

All experiments were performed with the same experimental program according to the gas composition and the melting time. The program consists of premelting, pre-reduction, reduction, and charging, and a final reduction after the charging. The design of the experiments is shown in Table 2. In the current study, twelve experiments were conducted to investigate the method’s applicability to evaluating optimal charging rates for the HPSR process. The tests differ by the type of iron ore charging (batch charging in the crucible and continuous charging), the system’s charging rate, and thus the absolute masses of charged oxygen per time during the premelting and feeding process. The value for the oxygen charged represents the iron bonded oxygen in the ore. Table 2 also shows the experiment names with the respective quantities of iron ore charged as a batch in the crucible and continuous with the feeding system. The iron ore charging rate is directly adjusted via the powder-dosing instrument. The oxygen-charged ratio is calculated via the mass flow of iron ore and the chemical composition in Table 3. The bonded oxygen in the charged material was calculated as an absolute value in grams. The LOI (loss of ignition) was also included in the calculations to consider the lost mass due to heating in the reactor.

Carajas hematite iron ore (Table 3) was used in these studies for the experiments. Table 4 presents the grain size distribution of the ore. Furthermore, Table 5 displays the chemical composition of the steel crucible and the steel pin material. The gases that were used and their purities are listed in Table 6.

### 3.3. Description of the Operation

**Figure 5:**
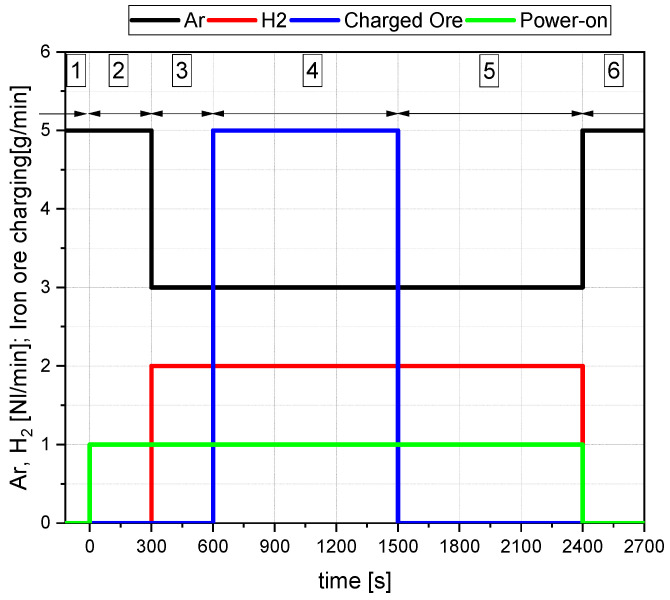
Representation of the performed process steps.

Before the facilities’ final assembly, the ore was dried for three hours at 130 °C in a drying closet. This drying was necessary to decrease the risk of material bridging during charging in the feeding system. The complete avoidance of the mentioned bridging was not possible. Experiments B50/C1.20 and B50/C0 were affected by it. Nevertheless, the experiments were not excluded from the test review, as valuable information for the evaluation could be gained from them. According to the experimental program, the mass of the dried material was charged in the crucible and feeding system (Table 2, column one). The testing procedure can be divided into six steps (Figure 5). Only argon is injected into the furnace in the first step without the supply of electrical energy. Afterward, the second phase (premelting) takes place. For that, the electrode was set to the contact position of the steel pin. In this position, the level indicator of the HGE was set to zero. Following the arc’s ignition, the electrode was raised to a height of 20 mm, and the iron oxide was melted and reduced via thermal decomposition towards magnetite. After the oxide was entirely melted down (normalized to 5 min of melting), the gas composition was changed to 3 L/min Ar and 2 L/min H_2_ for the pre-reduction phase. This third step was also carried out for 5 min. The following step, number four, comprises the charging of the iron ore material through the HGE into the plasma furnace and the ongoing reduction with 60% of Ar and 40% of H_2_ at the same time. Once the ore has been wholly fed, the post-reduction phase (five) follows until the measured off-gas shows a hydrogen content of 40%. Then, the reduction process is finished, and the arcing operation is stopped. Finally, in phase six, the furnace is flushed with pure argon or nitrogen to reduce the remaining hydrogen content below the critical level of 4 Vol%.

**Figure 6:**
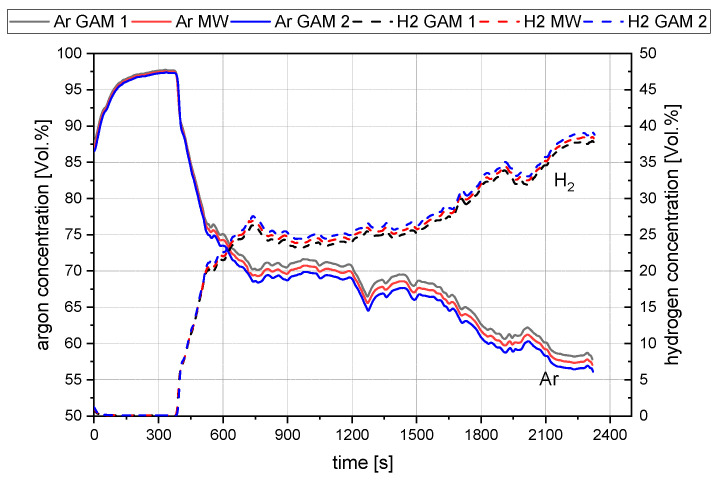
Evaluation for the mean values of trail B50/C1.16.

As mentioned in Section 3.1, two mass spectrometers were used to conduct the trials. All final results concerning the gas composition of this study are the mean values of the two measurements. Figure 6 shows the deviation between the two measurements (GAM1 and GAM2) for argon and hydrogen and the mean value of both lines (MW).

**Figure 7:**
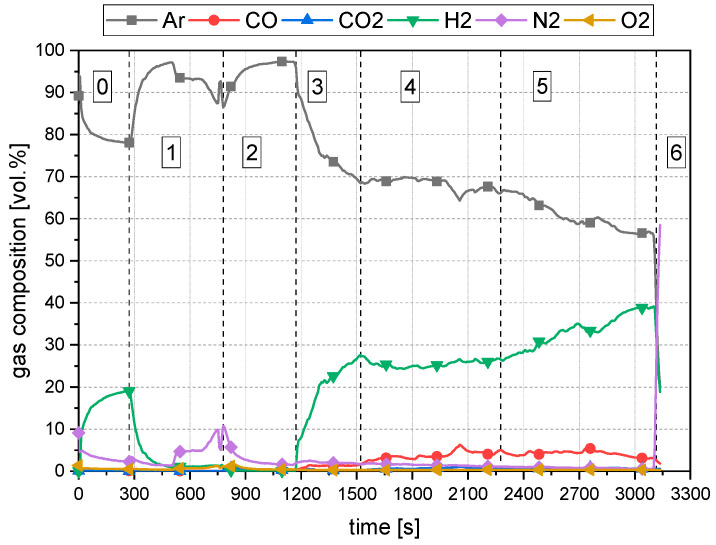
Representation of the performed process steps B50/C1.04.

The resulting gas analysis for the experimental program is shown in Figure 7. Phase 0 is not part of the testing procedure. In phase 0, the system’s tightness was checked to avoid any gas leakage during the trial. The drop of argon between phases 1 and 2 and the introduction of nitrogen in these phases occurs due to the gas supply stopping before starting the arcing process. This gas flow stop is necessary because during the ignition step, the distance between the HGE and the crucible is lowered to a point where ore would be blown away from the crucible on the full gas flow. Phases 3 to 5 show the trends for the reduction process with hydrogen. In phase 6, the applied nitrogen purging can be seen due to the sudden increase in the nitrogen content.

**Figure 8:**
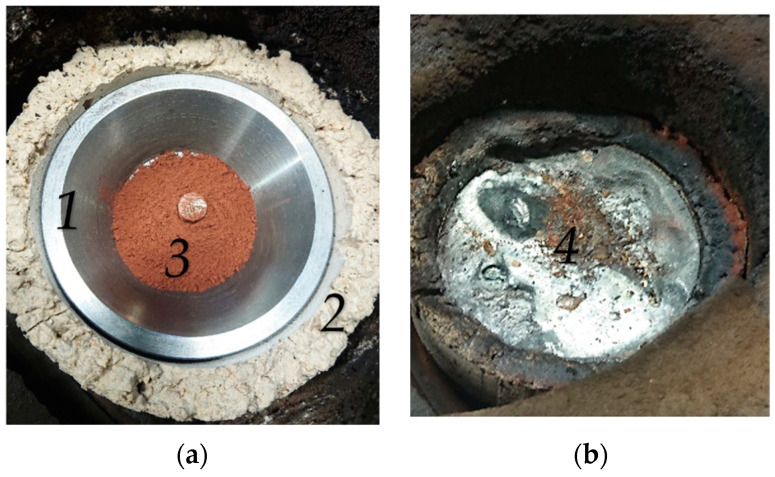
Crucible before (**a**) and after (**b**) Trial B50/C1.04.

The left photo in Figure 8 shows a view of the crucible (1) with the protective refractory ring (2) and with the charged ore batch (3) before the trial B50/C1.4. After the experiment, the right photo presents the crucible with the solidified molten material (4). It is evident that the melt has formed a more or less flat surface. The reason is that the crucible’s sidewalls were melted down. The total volume of the molten material is composed of the molten and reduced iron ore, the ignition pin, and the melted sidewalls of the crucible. A small amount of non-melted or reduced ore remains in the center on the surface. This discharged material originated from the HGE during the dismantling of the reactor. This amount is subtracted from the initially inserted ore mass (Table 3). Without this subtraction, the calculation of the reduction degree would result in excessively high values.

### 3.4. Methods for the Evaluation

The off-gas analysis is carried out by a mass spectrometer (MS) that detects hydrogen, nitrogen, carbon monoxide, carbon dioxide, oxygen, and argon. The output of the MS data in the software is in volume percent. At first, these values are converted into absolute volume values using argon. This can be done because argon does not take part in chemical reactions, and the volume flow is constant and precisely adjustable. The cycle time (t_cyc_) is defined by the measuring interval of each species of the MS. Between those measurements, a dwell time of around 50 ms is set. This leads to 6–7 s for the presented measures. Consequently, it is possible to calculate the absolute volume of argon (Equation (35)) by multiplying the cycle time with the time-specific volume flow.

(35) $$V_{Ar}=t_{cyc}*\;{\dot{V}}_{Ar}$$

(36) $$V_{CO_{2}}=c_{V,CO_{2}}\frac{V_{Ar}}{c_{V,Ar}}$$

(37) $$V_{\mathrm{CO}}=c_{V,\mathrm{CO}}\frac{V_{Ar}}{c_{V,Ar}}$$

(38) $$V_{{H_{2}}_{out}}=c_{V,H_{2}}\frac{V_{Ar}}{c_{V,Ar}}$$

$V_{CO}$_,_$V_{CO_{2}}$,$V_{{H_{2}}_{out}}$—Volume of CO, CO_2_, H_2_ in the exhaust gas for a measuring cycle [Nl]

$c_{V,CO}$_,_$c_{V,CO_{2}}$_,_$c_{V,H_{2},\;}\;c_{V,Ar}$—Gas concentration of CO, CO_2_, H_2_, and Ar in exhaust gas [vol.%]

${\dot{V}}_{Ar}$—Volume flow of Ar [Nl/min]

$V_{Ar}$—Volume of Ar for a measuring cycle [Nl]

$t_{cyc}$—Time of a measuring cycle [min]

Consequently, the volume of produced steam is calculated from the difference between the injected hydrogen volume and the volume of H_2_ in the off-gas.

(39) $$V_{H_{2}O}=\left({\dot{V}}_{{H_{2}}_{in}}*t_{cyc}\right)-V_{{H_{2}}_{out}}$$

${\dot{V}}_{{H_{2}}_{in}}$—Volume flow of H_2_ in the gas supply [Nl/min]

$V_{H_{2}O}$—Volume of the produced H_2_O in the measuring cycle [Nl]

The mass of oxygen carried out by carbon monoxide, carbon dioxide, and steam is calculated based on the evaluated volumes. The mass of removed oxygen of the previous period is also considered.

(40) $$m_{O}=m_{O,V}+\sum \frac{V_{CO}+2*V_{CO_{2}}+V_{H_{2}O}}{V_{m}}*M_{O}$$

$m_{O}$—Mass of Oxygen removed [g]

$m_{O,V}\;$—Mass of Oxygen removed in the previous periods [g]

$M_{O}=16\;g/\mathrm{mol}$—Molar mass of oxygen [g/mol]

$V_{X}$—Volume of the species X (CO, CO_2_, H_2_O) [Nl]

$V_{m}=22.41\;l/\mathrm{mol}$—Molar volume according to standard conditions [l/mol]

**Figure 9:**
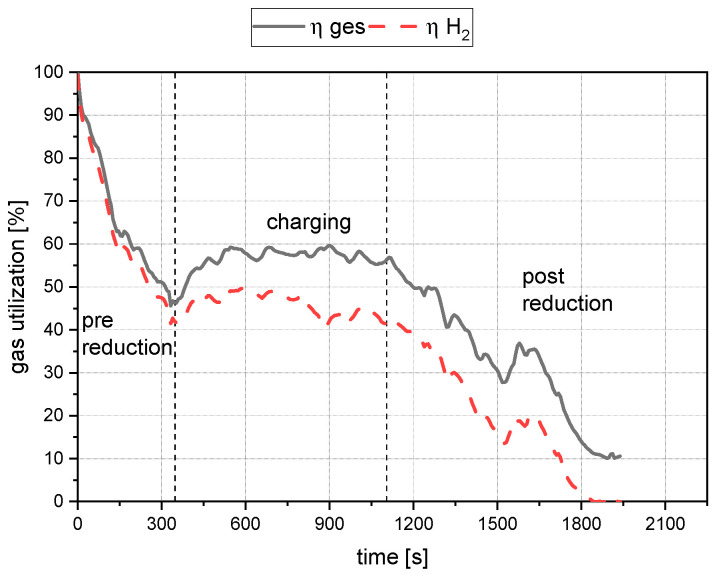
Total gas utilization and hydrogen gas utilization for trial B50/C1.04.

The species CO, CO_2_, and H_2_O, are used for X. In the case of CO_2_, the multiplication by two must be performed because carbon dioxide binds two oxygen atoms to itself. The carbon for this process originates mainly from the oxidation of the cathode. The gas utilization rate is calculated from the oxygen removing species’ quotient and the injected hydrogen volume. Figure 9 shows the evaluation of the gas utilization for trial B50/C1.04. The dashed lines mark the start and the end of the charging process.

(41) $$\eta_{H_{2}}=\frac{V_{H_{2}O}}{V_{H_{2}}+V_{H_{2}O}}$$

(42) $$\eta_{ges}=\frac{V_{H_{2}O}+V_{CO}+2V_{CO_{2}}}{V_{H_{2}}+V_{H_{2}O}}$$

$\eta_{H_{2}}$—Gas utilization rate for hydrogen

$\eta_{ges}$—Gas utilization rate for all oxygen carriers

**Figure 10:**
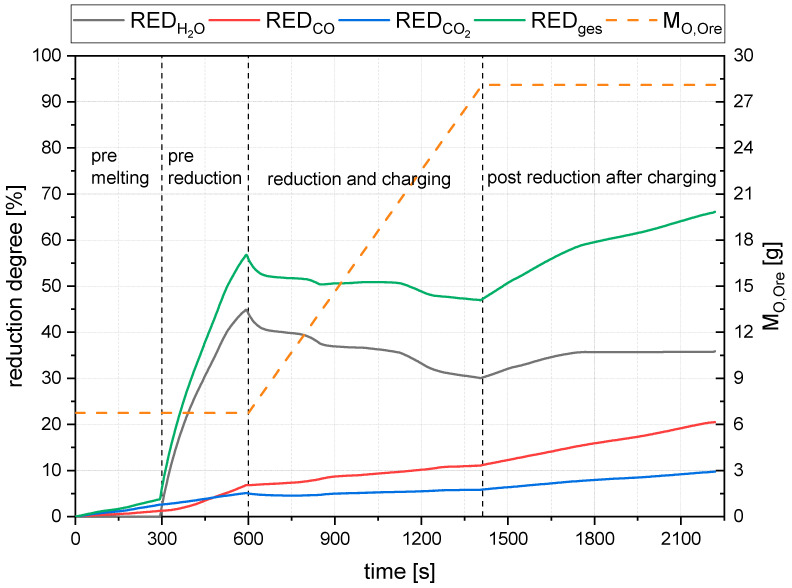
Reduction degrees for trial B25/C1.58.

Since the total utilization rate is calculated, as shown in Formula (42), there is no carbon entry in the denominator. This missing carbon value leads to the utilization rate reaching values higher than 100%. The reduction degree is calculated with the mass of oxygen removed by the individual species and the amount of oxygen bound to the iron ore in the reactor. Therefore, in experiments with continuous charging, the reducible oxygen content of the iron ore present in the reactor is changed during the trial. See the trend of M_O,Ore_ in Figure 10. The more ore is charged, the more oxygen is in the reactor via time. Thus, a change in the current oxygen content needs to be considered. Figure 10 shows the evaluated degrees of reduction for the formation of water (43), carbon monoxide (44), and carbon dioxide (45), and the total degree of reduction (46) for trial B25/C1.58.

(43) $$RED_{H_{2}O}=\frac{m_{O,H_{2}O}}{m_{O,Ore}}*100\%$$

(44) $$RED_{CO}=\frac{m_{O,CO}}{m_{O,Ore}}*100\%$$

(45) $$RED_{CO_{2}}=\frac{m_{O,CO_{2}}}{m_{O,Ore}}*100\%$$

(46) $$RED_{ges}=RED_{H_{2}O}+RED_{CO}+RED_{CO_{2}}$$

$RED_{H_{2}O},RED_{CO_{2}},RED_{CO}$—Reduction degree with H_2_, CO, and C [%]

$m_{O,Ore}$—Oxygen mass in ore [g]

$RED_{ges}$—Total reduction degree as a sum of RED [%]

The reduction rate r (Equation (47), corresponding to (33) and (34)) describes the conversion of the process as a time- and surface-dependent value. The reduction constant represents the relationship with the partial pressures of hydrogen and water vapor. Nagasaka et al. [35] determined for 1673 K:

(47) $$r=k_{a}\left[H_{2}\right]\left(P_{H_{2}}-\frac{P_{H_{2}O}}{K_{H}^{\prime }}\right)=1,6*10^{-6}\left(P_{H_{2}}-\frac{P_{H_{2}O}}{K_{H}^{\prime }}\right)$$

(48) $$k_{a}=\frac{r\prime }{\left(P_{H_{2}}-\frac{P_{H_{2}O}}{K_{H}^{\prime }}\right)*A}$$

r’—Oxygen removal rate [kg oxygen/s]

A—Area of the focal spot [m^2^]

**Figure 11:**
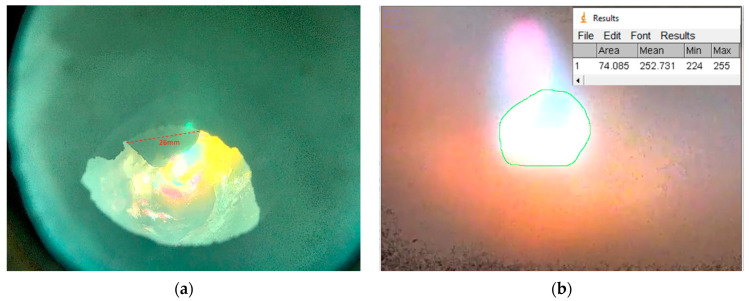
Evaluation method for the reaction surface. Calibration of the setup to determine the 26 mm outer diameter was taken at the start of the trial (**a**). Measurement of the surface area (A) during reduction, zoomed in and varied contrast (**b**).

Equation (47) is transformed (Equation (48)) and used to evaluate the r’ value in the reduction phase. Comparing this value for different settings should lead to the desired information about the kinetics. The k_a_ value can only be obtained for a defined surface area of the reduction (A [m^2^]). To evaluate the surface area A of the reduction zone, the camera system took images of the reactor interior. This evaluation should improve the quality of the factor for k_a_ compared to the evaluation by Nagasaka et al. [35] due to the determination of the specific surface. The hollow electrode has an outer diameter of 26 mm and is used as a reference for measuring the dimensions of the plasma focal spot. With “ImageJ”, an image processing program developed at the National Institutes of Health, the scale of the image has been defined so that the measurement result (the focal spot area) is displayed in square millimeters, see Figure 11. Accurate measurement results can be achieved by zooming in and varying the contrast of the images.

For evaluating the $k_{a}\prime$ value, the following values in Table 7 are considered or estimated.

(49) $$\mathrm{GOD}=\frac{x_{CO_{2}}+x_{H_{2}O}}{x_{CO_{2}}+x_{H_{2}O}+x_{CO}+x_{H_{2}}}$$

GOD—Gas Oxidation Degree [-]

$x_{CO_{2}},x_{H_{2}O},x_{CO},x_{H_{2}}$—Gas quantity of CO_2_, H_2_O, CO and H_2_ [mol]

**Figure 12:**
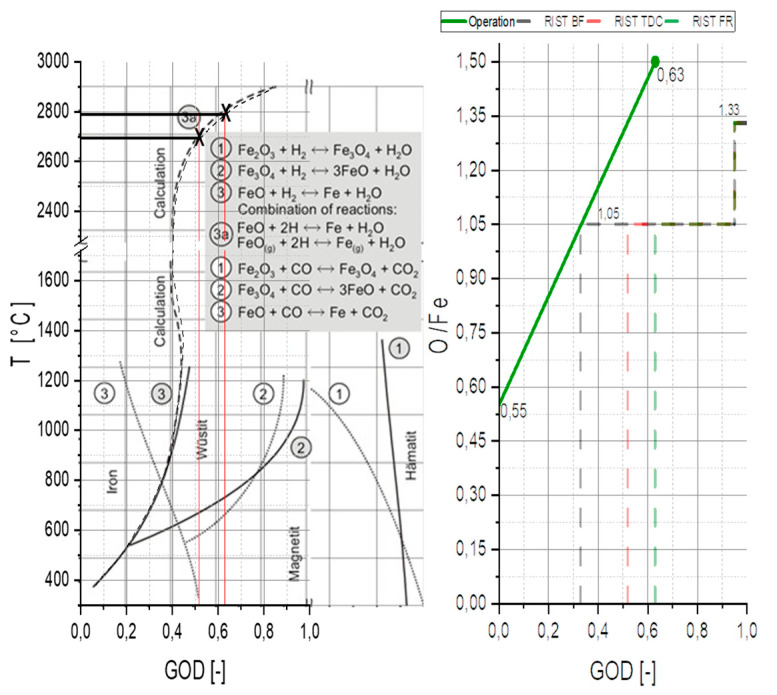
Stability fields of iron oxides in the Baur Glaessner (BG) Diagram (**left**) [4] combined with the operational points of trial B50/C1.56 shown in the RIST diagram (**right**), Operational field (OF) for Blast furnace (BF, grey dashed), OF for thermal decomposition (TDC, red dashed), and OF for full reduction (FR, green dashed) from hematite to metallic iron.

Figure 12 shows a RIST diagram (right) and a BG diagram (left). This combination can be used to estimate the average temperature of the plasma flame. To obtain this value, the wüstite point of the RIST diagram must be evaluated and transferred to the BG diagram. The temperature can be estimated according to the GOD for the evaluated point. For this estimation, it is assumed that the system is in equilibrium. This assumption can be made due to the flow’s high temperatures and high turbulence. In the RIST diagram, the operating line (green full) is defined by the reduction ratio (Figure 18) and the average gas utilization ratio (Figure 15) during the loading process of experiment B50/C1.56. The gas utilization ratio showed an average value of 0.63, and the reduction ratio reached 63%, corresponding to an O/Fe ratio of 0.55. The RIST diagram shows three dashed lines (gray, green, and red). These lines represent the respective versions of the RIST diagram for different assumptions of the operating parameters.

The gray line shows the blast furnace (BF) version of the RIST diagram. In this version, the reactor is assumed to be a countercurrent reactor. Due to the operating parameters in the HPSR plants, this line does not apply in the same way. The HPSR reactor is more of a mixed flow reactor. Therefore, two assumptions are possible. The first (red dashed) assumes that the ore that is introduced is decomposed by the thermal load and then reduced from the magnetite state to metallic iron. The second (green dashed) neglects thermal decomposition and reduces the hematite to metallic iron. Although the first option seems to be the logical choice, the second is the one that must be used. The ore is decomposed by the enormous thermal load when introduced into the reactor, but the free oxygen reacts directly with the hydrogen that is present. Thus, in defining the gas utilization in the exhaust gas, only the equilibrium state of the second variant can be observed in lines of mass spectrometry. By applying the described method for trial B50/C1.56, an average temperature for the reduction zone of ~2800 °C can be evaluated.

## 4. Results and Discussion

The selected results are presented in the following tables to show the essential data for the following discussion.

### 4.1. General Findings

**Figure 13:**
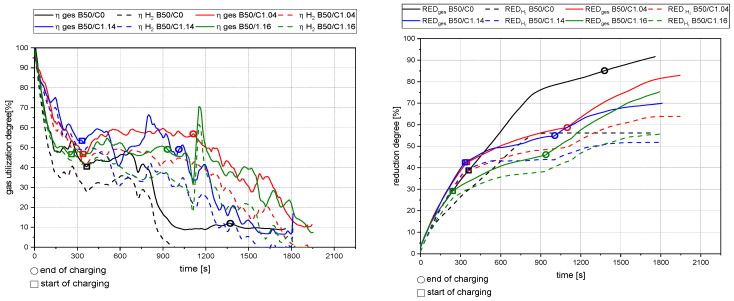
Gas utilization and reduction degree at 50 g batch and 50 g charged mass at 20 rounds per minute of the dosing unit.

**Figure 14:**
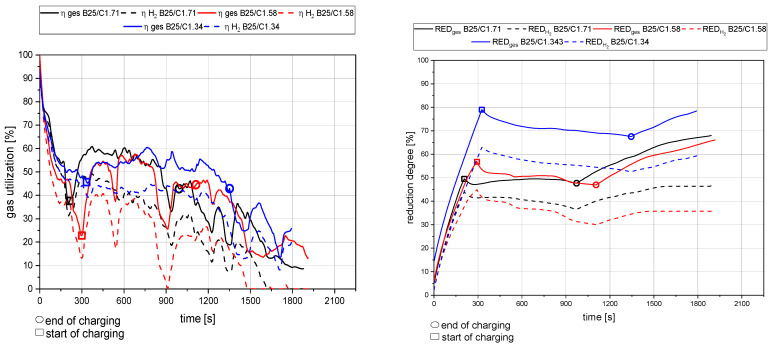
Gas utilization and reduction degree at 25 g batch and 75 g charged mass at 20 rounds per minute of the dosing unit.

**Figure 15:**
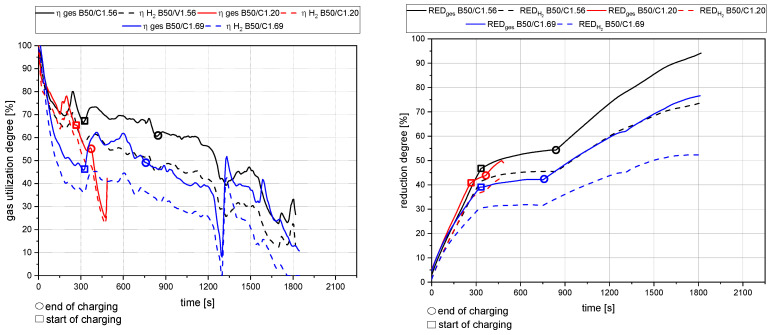
Gas utilization and reduction degree at 50 g batch and 50 g charged mass at 40 rounds per minute of the dosing unit.

**Figure 16:**
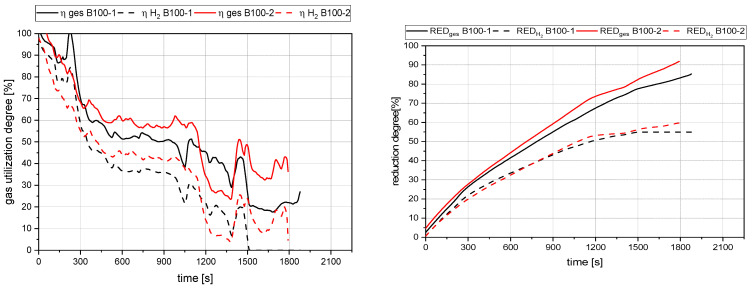
Gas utilization and reduction degree at 100 g batch and no charged mass.

Figure 13, Figure 14, Figure 15 and Figure 16 show the gas utilization degree graphs accompanied by the reduction degrees for different trials. A squared measurement point always marks the start of charging, and a circle indicates the end of charging. All data are presented in two ways: the solid lines are the full degrees and utilization, meaning the electrode’s carbon influence is considered; the dashed lines represent the degrees of reduction and utilization only for hydrogen. Three major phases can be observed for the trials with charged material.

1. The primary reduction of the batch material is indicated by a steep increase of the RD line (until the squared value);
2. The start of charging, indicated by a less steep phase of the RD graph (between the square and the circle);
3. The increase in the RD’s gradient due to the cessation of charging (after the circle).

Phase two is of significant interest for this investigation because the slope indicates the reduction kinetics. An ascending slope means that the introduced mass flow of oxygen via the ore is lower than the reduction process’s decreased amount. A descending slope means the exact opposite, and a steady RD line indicates an equilibrium between these two mass flows.

### 4.2. Detailed Discussion of the Results for All Three Phases

**Figure 17:**
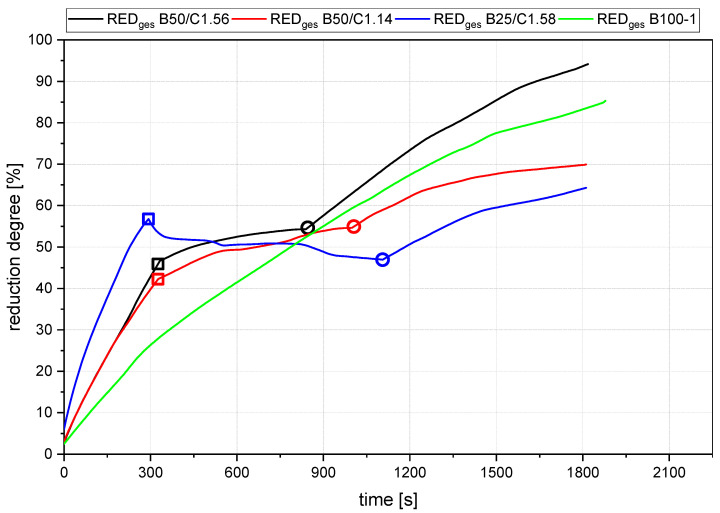
Comparison of reduction degrees of B50/C1.56, B25C1.58, B50/C1.14 and B100-1. Square: Start of Charging, Circle: End of Charging.

Figure 17 summarizes four representative curves for the reduction degree while operating at different setups. Out of the direct comparison of the curves in Phase 1, the pre-reduction step, the following significant points were observed:

- The rate of absolute removed oxygen per time in phase one seems similar for all trials. Therefore, the slope of the reduction degree differs at different starting masses of ore in the crucible. For lower amounts of batch-wise charged ore, the reduction degree increases faster.

**Figure 18:**
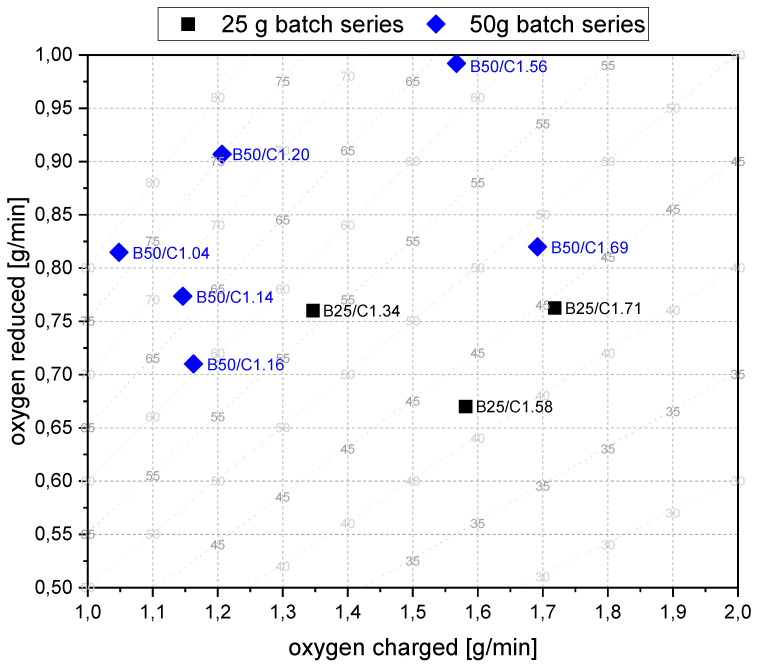
Comparison of the calculated oxygen removed via the oxygen supplied by the ore. Dashed lines in the background symbolize the equilibrium RED at infinite charging time.

**Figure 19:**
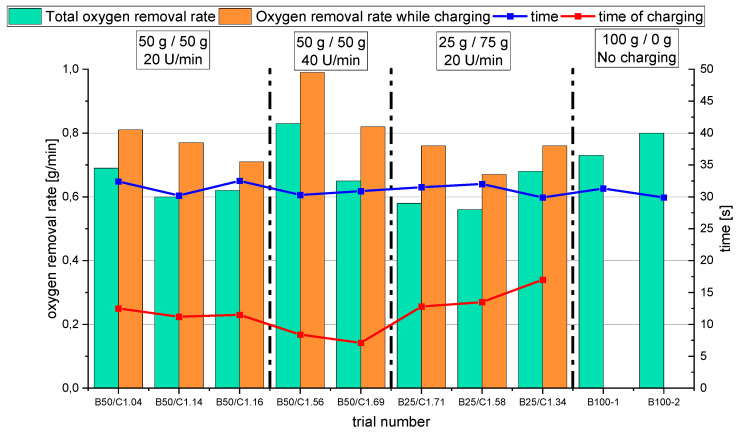
Comparison of the evaluated oxygen removal rates for the total trials (batch + charging) and for the time of charging.

For the detailed discussion of Phase 2, Figure 18 shows the oxygen that was removed via the oxygen introduced by the iron ore during the charging process. In the background, the reduction degree at constant parameters is shown in dashed lines. The reduction degree depicted is reached in a charged and reduced oxygen equilibrium. Table 8 shows the calculated values, which are the basis for this figure. For this step, the following statements were concluded:

- The slope of the reduction degree is directly linked to the charging rate. A higher/lower amount of oxygen is introduced at different charging rates. Therefore, the reduction degree changes until the equilibrium between the introduced and removed oxygen is reached. For higher amounts of charged ore, lower equilibrium reduction degrees are observed.
- For the B50 trials, an optimum reduction rate of 1 g/min could be evaluated at 1.56 g/min of introduced oxygen. At B25 tests, this optimum is not reached. A possible cause could be the higher reduction degree achieved in the first phase, which means the absolute amount of oxygen is lower; therefore, the kinetics are partially inhibited. This finding needs further investigation. Nevertheless, the demonstrated method seems to be able to compare and evaluate the optimal conditions for the reduction rate of the process.
- Comparing the reduction rates of the total trial and the charging time in Figure 19, the oxygen removal while charging is higher than the compared total rate. This means the reduction happens faster while charging compared to the batch trial.

For the third phase, the post-reduction, the following findings can be stated:

- The reduction rates and the accompanying reduction degrees vary for all trials. Therefore, pre-reduction and charging procedures can influence the final phase’s starting point and the reduction’s progression. A definitive statement is not possible due to the lack of data from systematic investigations. Further trials need to be carried out using a suitable experimental program
- In addition, the final reduction degree differs over a wide value range. The influencing factors of this phenomenon also require further investigations.

## 5. Conclusions and Outlook

The primary purpose of this work was to develop a method to compare the complex reduction kinetics system for different parameters in the HPSR process. The process’s theoretical thermodynamic and kinetic basics were summarized to reach this goal. Based on this background, the most important parameters that influence the process were defined, and the necessary equations for the comparison were demonstrated and explained.

The evaluation of the method showed four phases that influence the kinetics of the process. Each stage (premelting, pre-reduction, reduction and charging, and post-reduction) can be evaluated separately, and multiple different influences can be identified in each phase. The evaluation also showed that further experimental work is needed to understand each phase’s effects. For these other investigations, the phenomena in the charging phase are of the highest interest. The following bullet points show the suggested parameters for the examination.

### Planned Investigations for Part 2

- The influence of the specific amount of reducing agent in the gas phase needs to be investigated for further trials. This means that the optimal hydrogen rate for the steepest slope at the reduction degree curve needs to be found. This will lead to an optimal hydrogen gas rate for the pre-reduction phase.
- In addition, the influence of the specific gas rate (lower charging rate or higher total gas flow) is of significant interest in the description of the most favorable reduction conditions.
- The final reduction degree after the pre-reduction step could be an influencing factor for the ongoing reduction while charging. This could be because the rate of FeO-rich slag to produce iron influences the melting point of the bath. Therefore, the total size of the pool decreases at higher reduction degrees, and the kinetics for the reduction are lowered. Therefore, additional investigations need to be conducted.
- The possible connection between the oxygen supplied and oxygen reduced needs to be investigated.
- A suitable program for an accurate statistical evaluation needs to be defined.

## Figures and Tables

**Table 1:** Equipment of the HPSR laboratory facilities at the Chair of Ferrous Metallurgy.

Facility Manufacturer Info	Short Description
**Power Supply** inHouse, Leoben, Austria	The arc is a transferred DC arc with an average power input of ~5 kVA.
**SCR Power controller** inHouse, Leoben, Austria	Due to the implementation, the system can operate seamlessly from 1 to 16 kVA.
**Reactor and subdivisions** inHouse, Leoben, Austria	The hollow graphite electrode (HGE) has an inner diameter of 5 mm and a 24–26 mm outer diameter.
	The ignition pin has a 10 mm diameter and a height of 20 mm and is MIG welded to the crucible in two spots in the intersection area.
	The crucible has an outer diameter of 100 mm, a height of 35 mm, and can carry up to 100 g of ore at the start of the trial if the trial is operated in batch mode. The diameter of the flat base of the crucible is 54 mm.
	A refractory ring protects the bottom disc in case of a meltdown.
**Feeding System** LAMBDA Laboratory Instruments, Baar, Switzerland	Powder dosing system, using a powder distributor, which is coupled to a stepping motor.
**3x Process Gas** Linde Gas, Stadl-Paura, Austria	Argon and hydrogen are injected through the HGE into the arc zone to create ionized species. Nitrogen is used as a purging gas.
**3x Flow Controller** Bronkhorst High-tech B.V, AK Ruurlo, The Netherlands	Mass flow controllers type EL-FLOW PRESTIGE FG were utilized.
**2x Mass Spectrometry** Pfeiffer Vacuum Technologies, Aßlar, Germany	For the analysis of the off-gas, two mass spectrometers (MS Type GAM200) were used.
**Off Gas Cleaning System** inHouse, Leoben, Austria	Dedusting and dehumidification were ensured through the system.
**Camera System** Pieper GMBH, Berlin, Germany	The camera system Axis-Q1775 was used.

**Table 2:** Design of experiments of the trials at the HPSR laboratory facilities.

Denomination of the Experiment [–]	Iron ore Distribution Crucible/Continuous Feeding [g]	Continuous Charged Iron Ore [g]	Mass Flow of Iron Ore [g/min]	Oxygen Charged during Continuous Ore Feeding [g/min]
B50/C0	50/50	X	X	0
B50/C1.04	50/50	49.4	3.94	1.04
B50/C1.14	50/50	46.1	4.11	1.14
B50/C1.16	50/50	46.5	4.03	1.16
B50/C1.20	50/50	8.8	4.96	1.20
B50/C1.56,	50/50	48.6	5.76	1.56
B50/C1.69	50/50	46.5	6.55	1.69
B25/C1.37	25/75	73.6	4.33	1.34
B25/C1.58	25/75	75.1	5.54	1.58
B25/C1.71	25/75	75	5.88	1.71
B100-1	100/0 (Reference)	X	X	0
B100-2	100/0 (Reference)	X	X	0

**Table 3:** Chemical composition of the Carajas iron ore.

No	Element	[wt.%]
1	Fe_2_O_3_ ^1^	92.83
2	FeO	1.07
3	Total Fe	65.81
4	SiO_2_	1.694
5	Al_2_O_3_	1.01
6	Manganese	0.17
7	Phosphorus	0.057
8	Total sulfur	0.014
9	LOI ^2^	2.79

^1^ Calculated value after analysis by wet-chemical analysis. ^2^ Loss of ignition.

**Table 4:** The grain size distribution of Carajas iron ore.

Mesh Size [μm]	Fraction [wt.%]	Cum [wt.%]
63–125	50	50
25–63	50	100

**Table 5:** Chemical composition of ignition pin and steel crucible.

Element	Unit	C	Si	Mn	P	S	Cr	Mo	Ni	Al	Cu
Steel crucible	(wt.%)	0.178	0.261	1.325	0.009	0.005	0.083	0.031	0.168	0.027	0.179
Ignition pin	(wt.%)	0.441	0.217	0.85	0.008	0.028	0.985	0.162	0.085	0.021	0.116

**Table 6:** Qualities of the gases used for the trials.

Gas	Purity	Remarks
H_2_	5.0	-
Ar	5.0	O_2_, N_2_, H_2_O ≤ 5ppm
N_2_	5.0	O_2_, H_2_O ≤ 5ppm

**Table 7:** Description of the methods for the evaluation of the parameters for the calculation of the k_a_ value.

Parameter	Type of Evaluation	Description of the Method
r	measurement and calculation	The hydrogen content in the off-gas is measured, and according to Equation (40), the removed oxygen is calculated
P_H_2__	measurement	The mass spectrometry measures the value during the trial
P_H_2_O_	measurement and calculation	The mass spectrometry measures the hydrogen content, and the partial pressure of the water vapor is calculated according to the missing hydrogen of Equation (39)
K’_H_	set to a fixed value	The value is set to the gas utilization degree. Further explanations follow (Figure 12)
A	video, estimation, and measuring	The Area is measured via an image processing program from images of the reactor’s interior shown in Figure 11b

**Table 8:** Comparison of evaluated rates for oxygen removal of all conducted trials.

Trial [-]	r_O,red_ while H_2_-Supply [g/min]	H_2_ Flow [min]	r_O,red_ While Charging [g/min]	Charging [min]
B50/C0	0,40	29.4	X	X
B50/C1.04	0.68	32.4	0.81	12.5
B50/C1.14	0.60	30.2	0.77	11.2
B50/C1.16	0.62	32.5	0.71	11.5
B50/C1.20	0.84	8.1	0.91	1.8
B50/C1.56	0.83	30.3	0.99	8.4
B50/C1.69	0.65	30.9	0.82	7.1
B25/C1.34	0.68	29.9	0.76	17.0
B25/C1.58	0.56	32.0	0.67	13.5
B25/C1.71	0.58	31.5	0.76	12.8
B100-1 (ref)	0.72	31.3	X	X
B100-2 (ref)	0.80	29.9	X	X

## Abbreviations

3P	3 Phases
AC	Alternating Current
BF	Blast Furnace
BFP	Backflow preventer
BG Diagram	Baur Glaessner Diagram
DC	Direct Current
FIC	Flow indicator and controller
FR	Full Reduction
GOD	Gas Oxidation Degree
HGE	Hollow Graphite Electrode
HPSR	Hydrogen Plasma Smelting Reduction
LOI	Loss of Ignition
MIG Welding	Metal Inert Gas Welding
MS	Mass spectrometry
MW	Mean Value (Mittelwert)
OF	Operational Field
PI	Pressure indicator
RD, RED	Reduction Degree
SCR	Silicon Controlled Rectifier
TDC	Thermal Decomposition
**Formula Characters**	
${\dot{V}}_{{H_{2}}_{in}}$	Volume flow of H_2_ in the gas supply [Nl/min]
${\dot{V}}_{Ar}$	Volume flow of Ar [Nl/min]
$V_{X}$	Volume of the species X (CO, CO_2_, H_2_O) [Nl]
$E_{h}$ $,\;E_{m}$ $,\;E_{w}$	Activation energy [kJ/mol]
$K_{h}$ $,\;K_{m}$ $,\;K_{w}$	Equilibrium constants of Formulas (26) to (28)
$K_{H}^{\prime }$	Partial pressure ratio (^p^_H_2_O_)/(^p^_H_2__) in equilibrium
$M_{O}=16\;g/\mathrm{mol}$	Molar mass of oxygen [g/mol]
$RED_{H_{2}O},RED_{CO_{2}},RED_{CO}$	Reduction degree with H_2_, CO, and C [%]
$RED_{ges}$	Total reduction degree as a sum of RED [%]
$S_{h}$ $,\;S_{m}$ $,\;S_{w}$	Specific surface [m^2^/mol]
$V_{H_{2}O}$	Volume of the produced H_2_O in the measuring cycle [Nl]
*V_Ar_*	Volume of Ar for a measuring cycle [Nl]
$V_{CO}$${,}_{\;}V_{CO_{2}}$, $V_{{H_{2}}_{out}}$	Volume of CO, CO_2_, H_2_ in the exhaust gas for a measuring cycle [Nl]
$V_{m}=22.41\;l/\mathrm{mol}$	Molar volume according to standard conditions [l/mol]
$c^{H_{2}}$ $,\;c^{H_{2}O}$	Concentration of hydrogen/water in the gas [mol/m^3^]
$c_{V,CO}$ ${,}_{\;}c_{V,CO_{2}}$ ${,}_{\;}c_{V,H_{2},\;}\;c_{V,Ar}$	Gas concentration of CO, CO_2_, H_2,_ and Ar in exhaust gas [vol.%]
$k_{0}^{h}$ $,\;k_{0}^{m}$ $,\;k_{0}^{w}$	Pre-exponential factor [m/s]
$m_{O,V}$	Mass of Oxygen removed in the previous periods [g]
$m_{O,Ore}$	Oxygen mass in ore [g]
$m_{O}$	Mass of Oxygen removed [g]
$n_{h}$ $,\;n_{m}$ $,\;n_{w}$	Quantity of hematite/magnetite/wüstite [mol_h/m/w_]
$p_{H_{2}}$ $,\;p_{H_{2}O},\;p_{O_{2}}$	Partial pressure of hydrogen, water, and Oxygen [Pa]
$t_{cyc}$	Time of a measuring cycle [min]
$x_{CO_{2}},x_{H_{2}O},x_{CO},x_{H_{2}}$	Gas quantity of CO_2_, H_2_O, CO, and H_2_ [mol]
$\eta_{H_{2}}$	Gas utilization rate for hydrogen
$\eta_{ges}$	Gas utilization rate for all oxygen carriers
ΔG	Gibbs free energy change [J/mol]
A	Area of the focal spot [m^2^]
k_a_[H_2_]	Reaction rate constant wüstite/H_2_ [kg oxygen/m^2^ s Pa]
k_Ch_	rate constant of the chemical reaction [mol/s]
R	Ideal gas constant [kJ/mol * K]
R	Reaction rate [kg oxygen/m^2^s]
r’	Oxygen removal rate [kg oxygen/s]
t	Time [s]
T	Reaction temperature [K]
β	mass transport coefficient [m/s]
GOD	Gas Oxidation Degree [-]
